# Cruciform Formable Sequences within *Pou5f1* Enhancer Are Indispensable for Mouse ES Cell Integrity

**DOI:** 10.3390/ijms22073399

**Published:** 2021-03-26

**Authors:** Yu Yamamoto, Osamu Miura, Takashi Ohyama

**Affiliations:** 1 Department of Biology, Faculty of Education and Integrated Arts and Sciences, Waseda University, Shinjuku-ku, Tokyo 162-8480, Japan; yuuneaze@akane.waseda.jp; 2 Major in Integrative Bioscience and Biomedical Engineering, Graduate School of Science and Engineering, Waseda University, Shinjuku-ku, Tokyo 162-8480, Japan; miu36@asagi.waseda.jp

**Keywords:** inverted repeat (IR) sequence, cruciform, super-enhancer, eRNA, mouse ES cells, *Pou5f1* (*Oct3/4*), *Sox2*, *Nanog*, *Klf4*, *Esrrb*

## Abstract

DNA can adopt various structures besides the B-form. Among them, cruciform structures are formed on inverted repeat (IR) sequences. While cruciform formable IRs (CFIRs) are sometimes found in regulatory regions of transcription, their function in transcription remains elusive, especially in eukaryotes. We found a cluster of CFIRs within the mouse *Pou5f1* enhancer. Here, we demonstrate that this cluster or some member(s) plays an active role in the transcriptional regulation of not only *Pou5f1*, but also *Sox2*, *Nanog*, *Klf4* and *Esrrb*. To clarify in vivo function of the cluster, we performed genome editing using mouse ES cells, in which each of the CFIRs was altered to the corresponding mirror repeat sequence. The alterations reduced the level of the *Pou5f1* transcript in the genome-edited cell lines, and elevated those of *Sox2*, *Nanog*, *Klf4* and *Esrrb*. Furthermore, transcription of non-coding RNAs (ncRNAs) within the enhancer was also upregulated in the genome-edited cell lines, in a similar manner to *Sox2*, *Nanog*, *Klf4* and *Esrrb.* These ncRNAs are hypothesized to control the expression of these four pluripotency genes. The CFIRs present in the *Pou5f1* enhancer seem to be important to maintain the integrity of ES cells.

## 1. Introduction

Genomes contain many unusual DNA structures, including cruciform structures, left-handed DNA helices, curved DNA structures, triplex structures, and G-quadruplex (G4) structures. Among them, cruciform structures have the longest history of study. In 1955, two years after Watson and Crick revealed the right-handed double helical structure of DNA, Platt proposed the presence of cruciform structures [1]. Cruciforms can be formed where a given DNA has a complete inverted repeat sequence, which reads the same from 5′ to 3′ in either strand, or an incomplete inverted repeat (IR) that contains an appropriate length of spacer DNA between the repeating units. Numerous studies have been performed to understand the functional significance of cruciform and/or IR sequences. Although their implications in DNA replication [2,3,4], transcription [5,6,7,8,9,10,11], and recombination [12,13,14,15] have been suggested, their functional or mechanistic role in each process remains elusive.

To gain insight into the biological role of cruciform structures, we recently performed genome-wide analyses of the *Escherichia coli* and *Saccharomyces cerevisiae* genomes, constructed comprehensive maps of IRs, and classified them depending on their structures and positions, as compared with gene positions. In *E. coli,* cruciform formable IRs (CFIRs) were significantly enriched in five regions, including the adjacent regions downstream of stop codon-coding sites and on and around the positions corresponding to mRNA ends. Furthermore, most of the CFIRs with a repeat unit length of ≥8 bp and a spacer size of ≤8 were found to be parts of the intrinsic terminators [10]. Regarding *S. cerevisiae*, the close vicinity of the DNA positions corresponding to poly(A) sites was the most statistically significant region of IR enrichment, and these IRs strongly correlated with the poly(A) signal. In addition, the majority of the IRs caused low nucleosome occupancy [11]. These studies suggested that in both organisms, the IRs actively participate in the mechanism of transcription termination. However, these studies could not suggest any plausible mechanism for the functions of IRs or CFIRs in the regulation of transcription initiation or modulation of transcription.

To our knowledge, no report has shown the relevance of IR(s) or CFIR(s) in the functions of enhancers. Using the mouse genome, we recently performed a screening of CFIRs and found that mouse *Pou5f1* (*Oct3/4*) has a CFIR cluster within its enhancer. Based on the FANTOM5 cap analysis of gene expression (CAGE) database [16,17], the *Pou5f1* enhancer produces enhancer RNAs (eRNAs), which are a signature of active enhancers [18,19,20]. Two types of eRNAs have been identified: those transcribed bidirectionally and those transcribed unidirectionally. The former is short and not adenylated, whilst the latter is long and adenylated [18,21,22]. Judging from the FANTOM5 CAGE database [16,17], the eRNAs transcribed from this enhancer belong to the former type. Another notable point is that the enhancer of *Pou5f1* was originally regarded as a “general” enhancer [23,24,25], but it was later found to be a part of a super-enhancer (SE) [26]. The SEs are defined as the regions where multiple enhancers are clustered together, and they are associated with genes involved in determining cell identity in both the physiological and pathological states [27].

To determine the function of the CFIR cluster present in the *Pou5f1* enhancer, we performed genome editing in which each of the CFIRs was replaced with the corresponding mirror repeat (MR). This alteration downregulated the *Pou5f1* transcription, but upregulated the eRNA transcription and *Sox2*, *Nanog*, *Klf4* and *Esrrb* transcription. The eRNAs are hypothesized to control the expression of these four pluripotency genes. A delicate transcriptional regulation network seems to exist among these genes, in which CFIRs apparently play an active role.

## 2. Results

**Figure 1:**
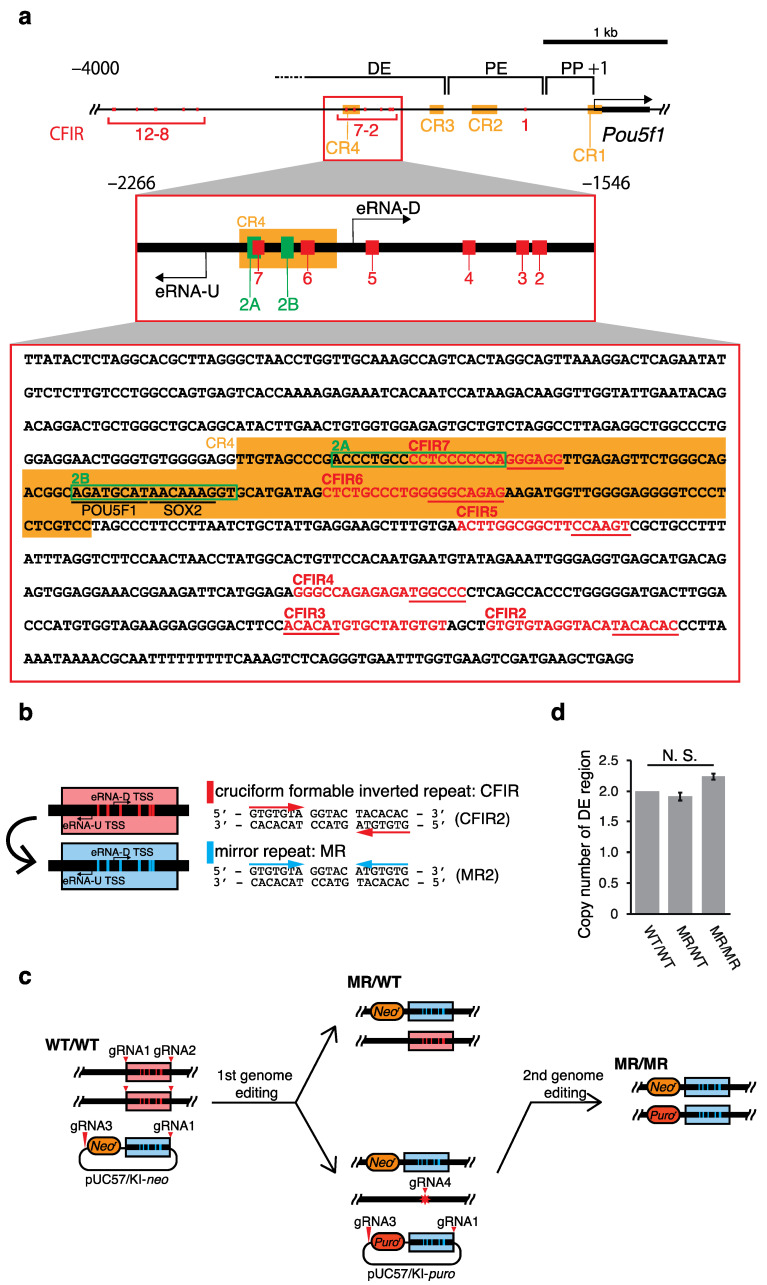
Regulatory region of *Pou5f1* and cruciform formable inverted repeats (CFIRs) as genome editing targets. (**a**) Map of the CFIRs and partial sequence of the distal enhancer (DE). The DNA sequence is from positions −2266 to −1546 relative to the TSS (+1) of *Pou5f1*. In the sequence, CFIRs are indicated with red characters, and the 2A and 2B sequences are enclosed within green rectangles. POU5F1 and SOX2 binding sites are underlined. The CR1 to CR4 regions (CR: conserved region) [29] are shaded in orange. The sequences underlined in red in the CFIR motifs were altered in the editing (Table 1). (**b**) Alteration of CFIRs into mirror repeats (MRs). CFIRs 2–7 were each altered to MRs 2–7, which maintained the GC content of each CFIR but removed its potential to form a cruciform. As an example, the alteration of CFIR2 to MR2 is shown on the right. (**c**) Pathway to establish genome-edited cell lines. The red triangles and star indicate target sites of guide RNAs (gRNAs) and repair junction, respectively. (**d**) Validation of the editing as examined by qPCR. Validation was performed by determining the copy number of DE, in which a part of *Pou5f1* was used for the copy number reference. Values are mean ± SD. The statistical analysis was performed using one-way ANOVA and Tukey’s post hoc analysis (*n* = 3).

Using our IR identifier CIRI created in-house [10], we screened the mouse genome and found that the upstream region of *Pou5f1* contains 12 IRs with repeat unit (R) lengths greater than or equal to 6 bp, spacer (S) lengths between 0 and 6 bp, and an entire IR length equal to or longer than 15 bp (i.e., IRs with R ≥ 6, S ≤ 6 and 2R + S ≥ 15) (Figure 1). Each of these IRs is thought to have the potential to form a cruciform [10,11], and thus they were named CFIR1 to CFIR12, respectively. Notably, six CFIRs (CFIR2 to CFIR7) are located in the region spanning from ~−2000 to ~−1600 relative to the transcription start site (TSS; +1) of *Pou5f1*. This region is within the distal enhancer (DE) of the gene (Figure 1a and Table 1). Among the 12 CFIRs, only CFIR2 to CFIR7 are located in this important region for the *Pou5f1* transcription. Thus, to determine whether this cluster has any function in *Pou5f1* transcription, using mouse ES cells, we substituted each of the six CFIRs with the corresponding MR sequence by 2H2OP method [28]-based genome editing, and examined the resulting effect on the transcription of the gene. This substitution did not change the nucleotide composition of each CFIR, but the resulting MR lost the cruciform-forming potential (Figure 1b). Finally, we established two cell lines, MR/WT (heterozygous editing) and MR/MR (homozygous editing) (Figure 1c). A sequence analysis of the region containing −2266 to −1546 (data not shown) and quantitative real-time PCR (qPCR) analyses confirmed that the substitution cassette was only present in the targeted region (Figure 1d).

### 2.1. Effects of the Genome Editing on Pou5f1 and eRNA Transcription

**Figure 2:**
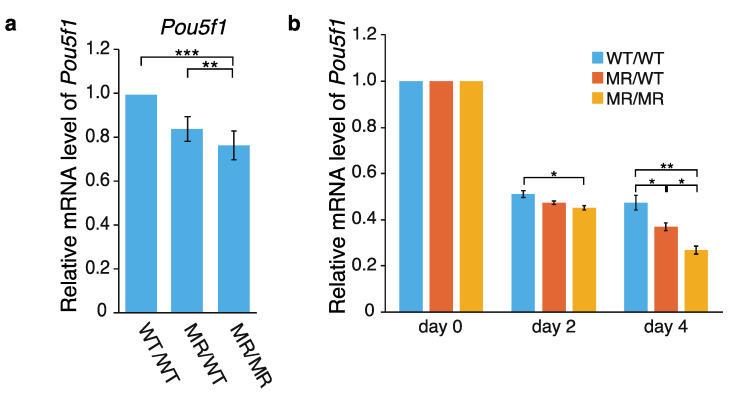
Effect of CFIR-to-MR editing on *Pou5f1* expression. Quantification was performed by qPCR. The level of *Pou5f1* mRNA in each cell line was normalized against that of *Gapdh*. (**a**) *Pou5f1* expression in the wild-type and genome-edited cells. The mean mRNA level in the wild-type WT/WT cells was set to 1.0, and the relative mRNA levels in the genome-edited MR/WT and MR/MR cells are shown. The values are represented as mean ± SD (*n* = 7). The statistical analysis was performed using one-way ANOVA and Tukey’s post hoc analysis. * *p* < 0.05; ** *p* < 0.01; *** *p* < 0.001. (**b**) Effects of leukemia inhibitory factor (LIF) depletion on *Pou5f1* expression. For quantification by qPCR, total RNAs were prepared immediately after LIF depletion (“day 0”), or two or four days after the depletion (“day 2” and “day 4”, respectively). In each cell line, the mean mRNA level at day 0 was set to 1.0 and relative transcript levels at day 2 and day 4 are shown. The values are represented as mean ± SD (*n* = 7). The statistical analysis was performed using one-way ANOVA and Tukey’s post hoc analysis. * *p* < 0.05; ** *p* < 0.01; *** *p* < 0.001.

Using WT/WT (wild-type), MR/WT and MR/MR cells, effects of the genome editing on *Pou5f1* transcription were first examined by qPCR. As shown in Figure 2a, the *Pou5f1* mRNA level was reduced in both the MR/WT and MR/MR cells, and the effect was larger in MR/MR than MR/WT. The transcription levels of the gene in MR/WT and MR/MR were ~85% and ~75% of that in the wild-type cells, respectively. Since the leukemia inhibitory factor (LIF) is required to maintain mouse ES cells in an undifferentiated state [30,31], we also examined the effects of LIF depletion. Although the transcription levels of *Pou5f1* were gradually reduced in all of the cell lines as time elapsed, the extents were higher in MR/WT and MR/MR cells, especially in the latter (Figure 2b). Regarding the data at day 4, the *Pou5f1* transcription levels in the former and the latter were ~80% and ~60% of that in the wild-type cells, respectively. Thus, the data shown in Figure 2a,b suggested that CFIRs 2–7 play an active role in *Pou5f1* transcription.

**Figure 3:**
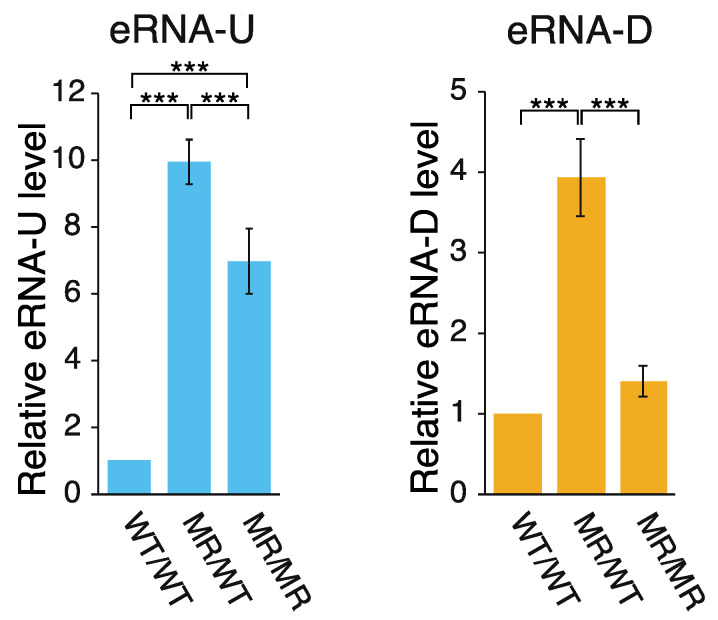
Effect of CFIR-to-MR editing on eRNA expression. The amount of eRNAs in each cell line was quantified by qPCR and normalized against that of *Gapdh* mRNA. The mean eRNA-U level (**left**) or mean eRNA-D level (**right**) in the WT/WT cells was set to 1.0 and the relative eRNA levels in MR/WT and MR/MR cells are shown. The values are represented as mean ± SD (*n* = 7). The statistical analysis was performed using one-way ANOVA and Tukey’s post hoc analysis. *** *p* < 0.001.

Active enhancers are transcribed to generate ncRNAs, which are referred to as eRNAs [18,19,20,32]. According to the FANTOM5 CAGE data [16,17], two eRNA species are transcribed bi-directionally within DE (Figure 1a). Therefore, to determine whether the alteration of CFIRs 2–7 to MRs 2–7 affected the transcription of these eRNA species, their relative transcription levels were analyzed by qPCR (Figure 3). Production of eRNA-U (eRNA transcribed toward upstream) was increased by ~10-fold and ~7-fold in the MR/WT and MR/MR cells, respectively, as compared to that in the wild-type cells. In the eRNA-U transcription, CFIRs 2–7 are all located upstream of the TSS for the RNA. Thus, it seems that some or all of the CFIRs 2–7 presumably play some repressive role in the eRNA-U transcription in the wild-type cells. On the other hand, the alteration of CFIRs 2–7 to MRs 2–7 had a smaller effect on eRNA-D (eRNA transcribed toward downstream) transcription: the activation was ~4-fold in MR/WT and only slight in MR/MR, as compared to the eRNA-D amount in the wild-type cells. Among CFIRs 2–7, only CFIRs 6 and 7 are located upstream of the eRNA-D TSS. Thus, the slightly different phenomena observed between eRNA-U and eRNA-D transcription may originate from the number of valid CFIRs in transcription: six in the former and two in the latter (Figure 1a). The result that the levels of eRNA-U and eRNA-D were both lower in MR/MR cells, as compared to those in MR/WT cells, may suggest the presence of a negative feedback mechanism in their production. Regardless of the hypothesis, the results suggest that at least some of CFIRs 2–7 have a negative influence on the transcription of the two eRNA species. Another important point is that the effects on transcription were opposite between *Pou5f1* and the two eRNA species.

### 2.2. Correlation between the Transcription of eRNA and That of Genes Controlled by a Super-Enhancer

**Figure 4:**
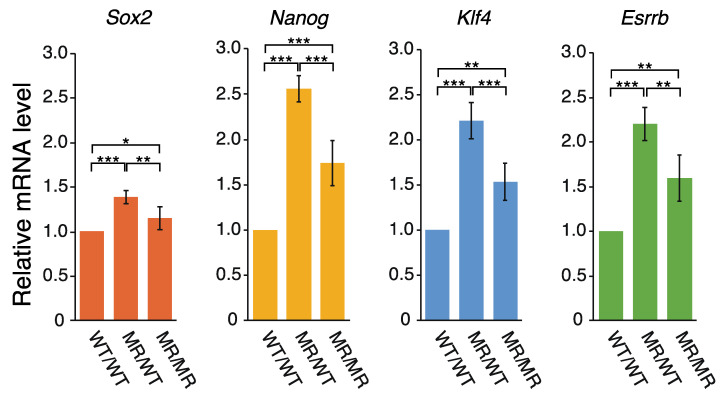
Effect on the expression of other genes regulated by the eRNAs. Quantification was performed by qPCR. The mRNA levels of *Sox2*, *Nanog*, *Klf4*, and *Esrrb* in each cell line were normalized against that of *Gapdh*. The mean mRNA level of *Sox2*, *Nanog*, *Klf4*, or *Esrrb* in the WT/WT cells was set to 1.0 and the mRNA levels relative to it in the MR/WT and MR/MR cells are shown. The values are represented as mean ± SD (*n* = 7). The statistical analysis was performed using one-way ANOVA and Tukey’s post hoc analysis. * *p* < 0.05; ** *p* < 0.01; *** *p* < 0.001.

The ~3 kb region upstream of *Pou5f1* reportedly acts as a super-enhancer [26], in which two notable sites were identified: one coincides with the 2B sequence and the other is within the proximal enhancer (PE) (Figure 1a). These are binding sites for transcription factors (TFs). The CFIR to MR alterations may affect the gene expression controlled by this super-enhancer. The relevant genes are *Pou5f1*, *Sox2*, *Nanog*, *Klf4*, and *Esrrb* [26]. We examined the effects of the genome editing on the transcription of *Sox2*, *Nanog*, *Klf4*, and *Esrrb*. Interestingly, the qPCR analysis showed that the transcription levels of these genes all exhibited the same changes in the following sense: the mRNA levels increased in both genome-edited cell lines, and those in MR/WT were higher than those in MR/MR (Figure 4). Extents of the transcription activation in the genome-edited cells were generally smaller for the four genes, as compared to the data for the eRNAs (Figure 3 and Figure 4), but the profiles of the changes were very similar to each other, especially between the four genes and eRNA-U. Although this study could not clarify whether eRNA-U or eRNA-D or both influenced the transcription of the four genes, the data shown in Figure 3 and Figure 4 strongly indicated that the eRNA(s) is (are) involved in the transcription of each of the four genes.

## 3. Discussion

### 3.1. Hypothetical Mechanism Underlying the Editing-Elicited Downregulation of Pou5f1 Expression

POU5F1 (OCT3/4) is a key TF for maintaining pluripotency in ES cells and iPS cells [33,34,35,36,37], and *Pou5f1* transcription is regulated by proximal promoter (PP), PE, and DE (Figure 1a) [23,24,25,34,38,39]. These three regions were determined by conventional approaches and some TF binding sites were identified, including the POU5F1 and SOX2 binding sites [40,41]. Analyses of the epigenetic features of these regions suggested that DNA methylation and histone modifications are strong regulators of *Pou5f1* expression [42,43,44,45,46]. However, no study has been performed to determine whether the CFIR cluster (CFIRs 2–7) is used in some regulation mechanism of *Pou5f1* expression.

A sequence homology analysis revealed that there are four conserved regions, named CR1 to CR4, in the upstream of the human, bovine and mouse *Pou5f1* TSSs [29]. In the mouse genome, only CR3 and CR4 are within DE, which contains the 2A and 2B sequences [41,47] and CFIRs 6 and 7 (Figure 1a). The 2B sequence harbors the POU5F1 and SOX2 binding sites [40,41]. The current study showed that the alteration of the cluster CFIRs 2–7 into MRs 2–7 within DE reduced the transcription of *Pou5f1*. Although the negative effects of MRs 2–7 were not large, the presence of the effect was obvious and ~20% transcriptional reduction occurred in MR/MR cells (Figure 2). Among the sequence alterations, those from CFIRs 6 and 7 to MRs 6 and 7 may be most relevant to the reduction, because they are in the vicinity of the 2A and 2B sequences.

The 2B sequence is located between CFIRs 6 and 7 and does not overlap with them (Figure 1a). Distances between 2B and CFIR7 and 2B and CFIR6 are 22 bp and 9 bp, respectively. In the alteration of CFIR6 to MR6, however, the upstream repeat unit (8 bp) and spacer sequence (3 bp) remained unchanged. Thus, the 17 bp 2B sequence has 20 or 22 bp intact flanking sequences on either side, suggesting that the transcriptional reduction of *Pou5f1* was not due to the sequence alteration itself, but rather to some other effect. One possible explanation is that the inhibition of the cruciform formation caused by the CFIR-to-MR alteration might have negatively influenced POU5F1 (and SOX2) binding to the 2B sequence, which may have impaired some mechanism in *Pou5f1* expression (POU5F1 binding-implicated positive feedback mechanism may exist). This scenario seems plausible if the protein binding induces DNA bending that entails unwinding. In this hypothetical case, a torsional stress imposed on the 2B region by the binding of POU5F1 (and SOX2) can be alleviated by the cruciforms formed on CFIR6 and/or CFIR7, which can rewind structurally unfavorable local DNA unwinding (melting), but MRs 6 and 7 cannot do this. Thus, this editing could have destabilized the binding of these TFs or even inhibited their binding. Many reports have described protein-induced DNA bending and unwinding [48,49,50,51,52]. After the *E. coli* promoter is wrapped around RNA polymerase (DNA is “intensely bent” on the enzyme), it also induces DNA unwinding [53]. These DNA unwinding phenomena are considered to be prerequisites for the subsequent step(s) to proceed. The same may be true for POU5F1 (and SOX2) binding.

The 18 bp 2A sequence is 100% conserved in the human and bovine alignment, and it shares 55.6% homology with the mouse 2A sequence [29]. Deletion of the sequence greatly reduced the *Pou5f1* expression in mouse cells [34,41], indicating that this sequence also plays a positive regulatory role in *Pou5f1* expression, at least in mice. Although CFIR7 partially shares a sequence with 2A, the alteration of CFIR7 to MR7 did not impair the 2A sequence itself (Figure 1a). Thus, transcriptional reduction of *Pou5f1* may be irrelevant to the function of the 2A sequence or it can be also explained in terms of protein-induced DNA bending and unwinding described above. However, to our knowledge, there has been no report that identified the 2A-binding protein [54]. Clearly, this issue needs further study.

### 3.2. eRNAs Transcribed from DE Are Presumably Implicated in the Transcriptional Regulation of Sox2, Nanog, Klf4, and Esrrb

The sequence alteration of CFIRs 2–7 to MRs 2–7 generated different effects on *Pou5f1* and eRNA transcription. Contrary to the *Pou5f1* transcription, this alteration upregulated eRNA transcription (Figure 2 and Figure 3), indicating that CFIRs 2–7 have some repressive effect on the eRNA transcription in mouse ES cells. Furthermore, the eRNA transcription profiles (Figure 3) correlated well with those of *Sox2*, *Nanog*, *Klf4*, and *Esrrb* (Figure 4). The upstream ~3kb region of *Pou5f1* act as an SE and it regulates the expression of these pluripotency genes [26].

**Figure 5:**
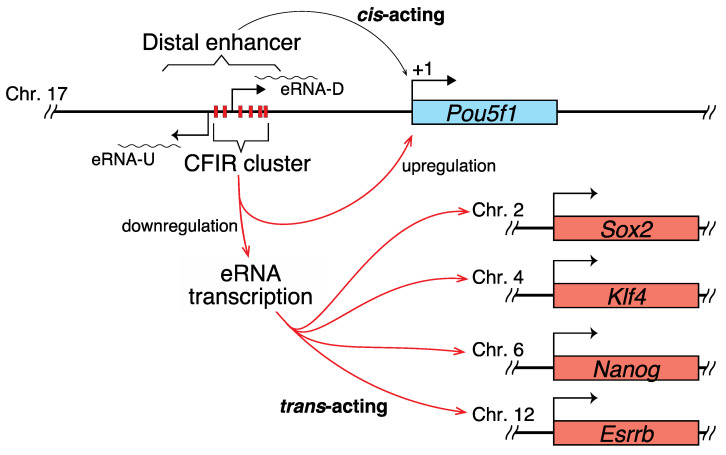
Hypothetical role of the CFIR cluster. The CFIR cluster focused on in the current study or some member of it is hypothesized to function as a modulator of the expression balance of the pluripotency genes *Pou5f1*, *Sox2*, *Nanog*, *Klf4*, and *Esrrb*, by upregulating *Pou5f1* expression while downregulating the other genes. In the downregulation mechanism for the latter, eRNA-U and eRNA-D, with expression that is also downregulated by the CFIR member(s), are strongly suggested to act as trans-acting molecules.

Two notable sites, which are binding sites for TFs, were identified in this SE: one coincides with the 2B sequence and the other is in the PE [26]. However, only the 2B sequence is relevant to the current study, and we hypothesized that CFIRs 6 and 7 may facilitate or stabilize POU5F1 (and SOX2) binding. On the other hand, stable binding of these TFs may be an obstacle in the transcription of eRNA-U and eRNA-D, which presumably act in trans to activate *Sox2*, *Nanog*, *Klf4*, and *Esrrb* expression (Figure 5). The different editing-caused effects on transcription observed between *Pou5f1* and the other genes may be explained in terms of the stoichiometry of their products in cells.

The genes that are involved in determining cell identity in both physiological and pathological states are generally regulated by SEs [27]. The SE focused on in the current study is known to influence the fate of pluripotent cells [26,55]. Considering this, the eRNA-U and eRNA-D expression levels may be strictly regulated by CFIRs to control the expression levels of *Sox2*, *Nanog*, *Klf4* and *Esrrb* and/or the balance between these gene products and that of *Pou5f1*, which may be important for maintaining the integrity of ES cells (Figure 5). Although some reports have suggested the mechanisms by which eRNAs activate transcription [56,57,58,59,60,61], the molecular mechanisms underlying the results obtained in the current study remain to be determined.

### 3.3. The Cluster of CFIRs Can Also Act as an Absorber of Negative Supercoils

We have mainly focused on CFIRs 6 and 7 to explain the phenomena caused by the editing. Finally, we must also discuss the possible functions of all six CFIRs. An important hint may be that cruciform formation can absorb the increased negative superhelicity that leads to DNA strand separation [62,63,64]. The six CFIRs exist in a small, 466 bp region spanning from the upstream end of CR4 to the downstream end of CFIR2. The negative superhelicity of this region may increase at some step preceding the eRNA and *Pou5f1* transcription or in the process of transcription, and should be generated upon nucleosome decomposition (detachment of histones). If this is the case, then most or all of these CFIRs may be used as “absorbers” of the increased negative superhelical density in the region, which seems beneficial in *Pou5f1* transcription, at least. Multiple or consecutive cruciforms may be further stabilized by inter-loop base-pairing, such as that found between the D-loop and TΨC-loop in tRNA [65,66].

## 4. Materials and Methods

### 4.1. Cell Culture and Transfection

The mouse ES cell line E14Tg2a [67] was maintained as described previously [68]. Briefly, the cells were maintained in G-MEM (Sigma-Aldrich, St. Louis, MO, USA), supplemented with 10% fetal bovine serum (Biosera, Nuaille, France), 0.1 mM 2-mercaptoethanol, 1x MEM non-essential amino acid solution (Thermo Fisher Scientific, Waltham, MA, USA), 1 mM sodium pyruvate (Thermo Fisher Scientific, Waltham, MA, USA), and 1000 units/mL of LIF (Cell Guidance Systems, Cambridge, UK), on gelatin-coated dishes without feeder cells at 37 °C in 5% CO_2_. For the 2H2OP method [28]-based genome editing, vectors and oligodeoxyribonucleotides (ODNs) were co-transfected into ES cells. Transfection was performed using Lipofectamine 3000 according to the manufacturer’s protocol (Thermo Fisher Scientific, Waltham, MA, USA), in which 250 ng of px330 vector, 250 ng of donor vector, 50 ng of ODN1 and 50 ng of ODN2 (ODN: oligodeoxyribonucleotide, Table S1) were used. Forty-eight h after the transfection, 200 µg/mL G418 (Enzo Life Sciences Inc., Farmingdale, NY, USA) or 0.1 µg/mL puromycin (Nacalai Tesque, Kyoto, Japan) was added to the culture for the selection. The cells were then cultured for two weeks. In the LIF depletion experiment, each cell line was cultured for two or four days after the depletion, and then total RNA was isolated.

### 4.2. Construction of Donor and Px330 Vectors

The pUC57/KI-neo plasmid, which carries the substitution donor DNA and the neomycin-resistance gene within the *Eco*RV site of pUC57, was prepared by Genewiz, Inc. (South Plainfield, NJ, USA). To generate pUC57/KI-puro, a DNA fragment encoding the puromycin-resistance gene in pSF-MinCMV-Puro (Oxford Genetics, Oxford, UK) was obtained by PCR, using the primers puro-fw-NcoI and puro-rv-XhoI (Table S1). The resulting fragment was inserted between the *Nco*I and *Xho*I sites of pUC57/KI-neo. The CRISPRdirect web tool (https://crispr.dbcls.jp/) [69] was used to design the gRNA sequences. After annealing the corresponding ODNs (Table S1), the resulting fragments were inserted within the *Bbs*I site of px330-U6-Chimeric-BB-CBh-hSpCas9 (Addgene, Watertown, MA, USA), to generate px330-gRNA1 to px330-gRNA4. The region containing gRNA expression cassettes in each construct was amplified by PCR, using the primers U6-gRNA-Fw-AflIII and U6-gRNA-Rv-HindIII or U6-gRNA-Fw-HindIII and U6-gRNA-Rv-AflIII (Table S1), and inserted into the *Afl*III site of px330-gRNA1. The resulting constructs px330-gRNA1-2-3 and px330-gRNA1-3-4 generated two sets of gRNAs: gRNA1, gRNA2 and gRNA3, and gRNA1, gRNA3 and gRNA4, respectively.

### 4.3. Sequence Analysis

The genomic DNA was purified using a conventional method. The sequence of the edited region was amplified by PCR with the primers KIcheck-fw and KIcheck-rv (Table S1). The resulting products were confirmed by Sanger sequencing, using a Big Dye Terminator v3.1 Cycle Sequencing Kit (Thermo Fisher Scientific, Waltham, MA, USA) according to the manufacturer’s protocol.

### 4.4. RNA Isolation and Reverse Transcription

Total RNA was isolated from the cells with a RNeasy Mini Kit (Qiagen, Venlo, The Netherlands) and QIAshredder (Qiagen, Venlo, Netherlands), and treated with RQ1 RNase-free DNase (Promega, Madison, WI, USA), according to the manufacturers’ protocols. The resulting sample was subjected to reverse transcription with ReverTra Ace (Toyobo, Osaka, Japan). Briefly, 1 µg of purified total RNA was used in the reaction in the presence of 5 pmol of oligo(dT)20 (Toyobo, Osaka, Japan), and 25 pmol of random primer (Toyobo, Osaka, Japan).

### 4.5. qPCR Analysis

The qPCR was performed using the primer sets shown in Table S1, with a StepOnePlus system (Thermo Fisher Scientific, Waltham, MA, USA) and THUNDERBIRD SYBR qPCR Mix (Toyobo, Osaka, Japan), according to Toyobo’s protocol. After the reverse transcription described above, reverse transcripts from 1.5 ng-equivalents (to quantify mRNAs) or those of 25 ng-equivalents (to quantify ncRNAs) of total RNA were subjected to PCR. For the gene copy analysis, 0.8 ng of genomic DNA was used. For qPCR, we used the following conditions: 95 °C, 2 min; 40 cycles of 98 °C, 10 sec and 68 °C, 1 min. To assess the specificity of the amplification, a melting analysis (from 60 °C to 95 °C with a 0.3 °C increment every 15 s) was performed at the end of the PCR cycles.

## Figures and Tables

**Table 1:** Sequences and structures of CFIRs 2–7.

Name	Position Relative to TSS (+1)		Structure (bp)			Sequence (Watson Strand) ^1^
	Start	End	Repeat Unit	Spacer	Total	
CFIR2	−1615	−1634	7	6	20	GTGTGTAGGTACATACACAC
						atgtgtg
CFIR3	−1639	−1655	6	5	17	ACACATGTGCTATGTGT
						tgtgta
CFIR4	−1710	−1727	6	6	18	GGGCCAGAGAGATGGCCC
						accggg
CFIR5	−1839	−1856	6	6	18	ACTTGGCGGCTTCCAAGT
						ggttca
CFIR6	−1925	−1943	8	3	19	CTCTGCCCTGGGGGCAGAG
						cccgtctc
CFIR7	−1992	−2007	6	4	16	CCTCCCCCCAGGGAGG
						ccctcc

^1^ Small characters under each CFIR indicate the sequence newly created by the editing, which generated the corresponding MR.

## Data Availability

Not applicable.

## Supplementary Materials

The following is available online at https://www.mdpi.com/article/10.3390/ijms22073399/s1, Table S1: Primers used in the study.
