# Supplementary material for: Cruciform Formable Sequences within Pou5f1 Enhancer Are Indispensable for Mouse ES Cell Integrity
Source: Int J Mol Sci. 2021 Mar 26;22(7):3399. doi: 10.3390/ijms22073399 (PMC8036336; doi:10.3390/ijms22073399)
Supplement: Supplementary file 1 [file ijms-22-03399-s001.pdf]

Table S1. Primers used in the study

Name	Sequence (5' to 3')
gRNA1-bottom	CACCGTTATACTCTAGGCACGCTTA
gRNA1-top	AAACTAAGCGTGCCTAGAGTATAAC
gRNA2-bottom	CACCGTGGTGAAGTCGATGAAGCTG
gRNA2-top	AAACCAGCTTCATCGACTTCACCAC
gRNA3-bottom	CACCGCCACAAAAGCGATTTCCGAG
gRNA3-top	AAACCTCGGAAATCGCTTTTGTGGC
gRNA4-bottom	AAACCAGGCGTGCCTAGAGTATAAC
gRNA4-top	CACCGTTATACTCTAGGCACGCCTG
KIcheck-fw	TTCTAAGAAGACTTGGGACTTCAGAC
KIcheck-rv	TCTTGATGGTAGTCTTCATCAGGTAG
puro-fw-NcoI	AATGCCATGGCATCTTGGACCATTAGCTCC
puro-rv-XhoI	ATGCCTCGAGTAGGGAGGGAAGAAAGTTTGAT
U6-gRNA-Fw-AflIII	GCTCACATGTGAGGGCCTATTTCCCATG
U6-gRNA-Fw-HindIII	ACCCAAGCTTGAGGGCCTATTTCCCATG
U6-gRNA-Rv-AflIII	GTGTACATGTCACGCGCTAAAAACGGACTA
U6-gRNA-Rv-HindIII	ACCCAAGCTTCACGCGCTAAAAACGGACTA
ODN-1	CAAAGTTGCTGTTAAGGACTGTATTATACTCTAGGCACGCGAGCGGTTAGGGCTAACCTGGTTGCAAAGCCAGTCACTAG
ODN-2	AAGTCTCAGGGTGAATTTGGTGAAGTCGATGAAGCCCTAACTGAGGCAGGAGAATTATCAGGAGTTCAAGGGCAGCTTGT
Copy-Num-ctrl-Pou5f1-fw	AAAGACACTGCTGAGGGCGA
Copy-Num-ctrl-Pou5f1-rv	AGACAGAAGCAGCAGCCAGT
Copy-Num-DE-fw	TGGGAGGTGAGCATGACAGA
Copy-Num-DE-rv	AGTCCCCTCCTTCTACCACATG
RT-Gapdh-fw	TTGGGCTACACTGAGGACCA
RT-Gapdh-rv	CCTGTTGCTGTAGCCGTATTCA
RT-Pou5f1-fw	GCAGATCACTCACATCGCCA
RT-Pou5f1-rv	AAGGTGTCCCTGTAGCCTCA
RT-Sox2-fw	CGGCAGCTACAGCATGATGC
RT-Sox2-rv	TCTGCGAGCTGGTCATGGAG
RT-Nanog-fw	GGATGAAGTGCAAGCGGTGG
RT-Nanog-rv	GGCTTCCAGATGCGTTCACC
RT-Klf4-fw	ATGCAGGCTGTGGCAAAACC
RT-Klf4-rv	TTTGCGGTAGTGCCTGGTCA
RT-Esrrb-fw	GCTGGACCTTTACCGAGCCA
RT-Esrrb-rv	CACCGCCTCCAGGTTCTCAA
RT-eRNA-D-fw	CGCTGCCTTTATTTAGGTCTTCCAACTA
RT-eRNA-D-rv	TCATGCTCACCTCCCAATTTCTATACA
RT-eRNA-U-fw	ACAATCCATAAGACAAGGTTGGTATTGA
RT-eRNA-U-rv	TTCAAGTATGCCTGCAGCCC
